# Birth Weight and Polycystic Ovary Syndrome in Adult Life: Is There a Causal Link?

**DOI:** 10.1371/journal.pone.0122050

**Published:** 2015-03-19

**Authors:** Stavroula A. Paschou, Dimitrios Ioannidis, Evangeline Vassilatou, Maria Mizamtsidi, Maria Panagou, Dimitrios Lilis, Ioanna Tzavara, Andromachi Vryonidou

**Affiliations:** 1 Department of Endocrinology and Diabetes, Hellenic Red Cross Hospital, Athens, Greece; 2 Department of Endocrinology and Diabetes, “Amalia Fleming” Hospital, Athens, Greece; 3 Endocrine Unit, Second Department of Medicine, “Attikon” University Hospital, Athens, Greece; University of Warwick – Medical School, UNITED KINGDOM

## Abstract

**Objectives:**

Several studies have demonstrated associations of birth weight with metabolic and reproductive abnormalities in adults. The aim of this study was to investigate the birth weight in women with PCOS and its correlation with clinical and biochemical characteristics of the syndrome.

**Materials and Methods:**

We studied 288 women with PCOS according to the NIH criteria and 166 women with normal cycle and without clinical hyperandrogenism. Birth weight and anthropometric characteristics were recorded, and levels of serum androgens, SHBG, insulin and fasting glucose were measured.

**Results:**

Birth weight data were available for 243/288 women with PCOS and age- and BMI-matched 101/166 controls. No differences were found (p> 0.05) in birth weight among women with PCOS and normal controls. Birth weight of PCOS women was negatively correlated with DHEAS levels (p = 0.031, r = -0.143) and positively correlated with waist circumference (p <0.001, r = 0.297) and body mass index (BMI) (p = 0.040, r = 0.132). Birth weight of controls was negatively correlated with SHBG levels (p = 0.021, r = -0.234). Women from both groups were further divided in 6 categories according to birth weight (A. <2.500 gr, B. 2.501-3.000 gr, C. 3.001-3.500 gr, D. 3.501-4.000 gr, E. 4.001-4.500 gr, F. > 4.500 gr). No statistically significant differences were observed in the distribution percentages between PCOS women and controls. (A. 7% vs 7.9%, B. 26.8% vs 20.8%, C. 39.1% vs 48.5%, D. 21.4% vs 20.8%, E. 4.9% vs 2%, F. 0.8% vs 0%), (in all comparisons, p> 0.05).

**Conclusions:**

Women with PCOS do not differ from controls in birth weight distribution. However, birth weight may contribute to subtypes of the syndrome that are characterized by adrenal hyperandrogenism and central obesity.

## Introduction

Birth weight can be considered as the “reflection” of endometrial life, representing in a large scale the maternal environment. It has been hypothesized that Polycystic ovary syndrome (PCOS) may have early origins in intrauterine life [1]. Evidence from experimental and clinical studies suggest that the endometrial environment may induce permanent changes in tissue structure or function favoring the development of PCOS in adult life. Prenatal androgen excess or deranged nutritional conditions during pregnancy may result in homeostatic adaptations of female fetuses through complicated mechanisms of fetal programming, which can lead to a more masculine and obese phenotype [2–3].

A few studies have been conducted to investigate a causative link between women’s birth weight and PCOS and results were inconclusive [4–8]. Some of them have demonstrated an association of low birth weight with hyperandrogenism and PCOS phenotype in adult life [4–6]. Furthermore, there are data showing that not only low but also increased birth weight may be associated with the presence of PCOS [5–6]. On the contrary, other large epidemiological studies did not show any association of birth weight with reproductive and metabolic abnormalities in PCOS patients, their female or their male relatives [7–8].

The aim of this study was to investigate birth weight in women with PCOS and its possible association with clinical and biochemical characteristics of the syndrome.

## Materials and Methods

### Patients and Controls

We studied 288 women with PCOS (25 ± 6.1 years) and 166 women (25.7 ± 6.4 years) as normal controls. Patients with PCOS were selected from the outpatient clinics of two centers (Hellenic Red Cross Hospital and “Amalia Fleming” Hospital). Control women were medical or dietology students or hospitals′ personnel.

Research has been approved by the Institutional Review Boards of the Hellenic Red Cross Hospital and "Amalia Fleming" Hospital in Athens, Greece and all clinical investigations have been conducted according to the principles expressed in the Declaration of Helsinki. Written informed consent was obtained from all participants.

We used the National Institute of Health (NIH) diagnostic criteria for PCOS patients; less than eight menses per year and a free androgen index (FAI) greater than 5 and/or clinical hyperandrogenism (presence of acne and/or hirsutism) [9]. Other causes of anovulation and hyperandrogenism were excluded with appropriate tests. Control women reported regular menstrual cycles and no clinical evidence of hyperandrogenemia.

Inclusion criteria for patients and controls: 1) women who were born full-term (38 0/7 till 41 6/7 weeks of gestation), 2) no known diabetes, hypertension or dyslipidaemia or any other medical or psychiatric illness and 3) no use of any medication including oral contraceptives, anti-androgens or metformin for at least three months prior to the study.

### Study protocol

Medical and reproductive history of PCOS patients and controls was recorded and physical examination was undertaken by endocrinologists. Birth weight and gestational age were self-reported for the majority of participants, while for the rest information was obtained from medical records. Nevertheless, in a previous study, it has been shown that there is a high degree of concordance between self-reported and actual birth weight [8].

Anthropometric measurements including weight, height and waist circumference were performed. Clinical hyperandrogenism was assessed by the presence of acne and/or hirsutism (modified Ferriman—Gallwey score > 8) [10].

Morning blood samples were drawn from all participants, after an overnight fast. Plasma glucose, insulin, total testosterone (TT), Δ4-androstenedione (Δ4A), dehydroepiandrosterone sulfate (DHEA-S) and sex hormone binding globulin (SHBG) were determined in the early follicular phase of the menstrual cycle. HOMA-IR (homeostatic model assessment-insulin resistance) was calculated by using the mathematic model HOMA-IR = glucose x insulin/405 (glucose in mg/dl) for the evaluation of insulin resistance [11].

### Assays

Plasma Glucose levels were measured by an enzymatic, colorimetric method in a Cobas Integra/400/700/800 autoanalyzer (Roche Laboratory Systems).

Serum insulin was measured by an immunoradiometric assay (IRMA, DIASource Immunoassays S.A.) with a sensitivity of 1 μIU/ml and intra- and interassay coefficients of variation of 2.1% and 6.5%, respectively. Serum SHBG levels were measured by an immunoradiometric assay (IRMA, Immunotech s.r.o.) with a sensitivity of 0.4 nmol/l and intra- and interassay coefficients of variation of 6.1 and 8.3%, respectively. TT was measured by radioimmunoassay (RIA, Cisbio Bioassays) with a sensitivity of 0.086 ng/ml and intra- and interassay coefficients of variation of 6% and 8.5%, respectively. Δ4A levels were measured by radioimmunoassay (RIA, DIASource Immunoassays S.A.) with a sensitivity of 0.03 ng/ml and intra- and interassay coefficients of variation of 4.5% and 9%, respectively. DHEA-S levels were measured by radioimmunoassay (RIA, Coat-A-Count DPC-Siemens) with a sensitivity of 1.1 μg/dl and intra- and interassay coefficients of variation of 5.3% and 8.1%, respectively.

### Statistical Analysis

Results are presented as mean ± SD for continuous variables. Results are presented as absolute numbers or percentages for categorical variables. Differences in continuous variables between groups were tested using the independent T-Test or Mann-Whitney U test, as appropriate. Differences in categorical variables between groups were tested using *χ* ^2^ test with Yates Correction. Pearson’s or Spearman’s Correlation was used to explore the association between pairs of continuous variables, as appropriate. All statistical analyses were performed using the Statistical Package for Social Sciences (SPSS 16.0, Inc, Chicago, IL, USA). A p value of <0.05 was considered statistically significant.

## Results

The clinical and biochemical characteristics of the study participants are shown in Table 1. Patients and controls were matched for age and BMI. Women with PCOS had significantly increased waist circumference (p<0.001), and HOMA-IR (p<0.001) compared to controls. They had also significantly increased levels of all androgens (Total Testosterone p<0.001, Δ4A p<0.001 and DHEA-S p<0.001) and significantly reduced levels of SHBG (p<0.001) compared to controls, as expected (Table 1).

**Table 1:** Comparison of important characteristics between patients with PCOS and controls.

	PCOS Patients (n = 288)	Controls (n = 174)	p value
**Age (years)**	**25 ± 6.1**	**25.7 ± 6.4**	**ns**
**BMI (kg/m** ^**2**^ **)**	**28.4 ± 7.3**	**27.2 ± 7.5**	**ns**
**Waist Circumference (cm)**	**89.7 ± 17.8**	**83 ± 16.3**	**< 0.001**
**HOMA-IR**	**3.27 ± 2.1**	**2.2 ± 1.5**	**< 0.001**
**TTesto (ng/ml)**	**0.89 ± 0.24**	**0.41 ± 0.14**	**< 0.001**
**Δ4A (ng/ml)**	**3.48 ± 1.3**	**1.8 ± 0.5**	**< 0.001**
**DHEAS (μg/dl)**	**295.5 ± 122.6**	**218.8 ± 87.7**	**< 0.001**
**SHBG (nmol/lt)**	**31.6 ± 13.3**	**51.5 ± 21.5**	**< 0.001**
**Birth Weight (gr)**	3243 ± 522 (*n = 243)	3212 ± 451 (*n = 101)	**ns**

BMI: body mass index; HOMA-IR: homeostatic model assessment-insulin resistance; TTesto: Total Testosterone; Δ4A: androstenedione; DHEAS: dehydroepiandrosterone sulfate; SHBG: sex hormone binding globulin.

Birth weight data were available for 243/288 PCOS women and for 101/166 controls. No differences in birth weight were found (p> 0.05) between PCOS women (3243 ± 522 gr) and normal controls (3212 ± 451 gr) (Table 2).

**Table 2:** Correlations of birth weight with important clinical and biochemical parameters of PCOS.

Patients	Birth Weight (gr)
**BMI (kg/m** ^**2**^ **)**	**p = 0.040** **r = 0.132**
**Waist Circumference (cm)**	**p < 0.001** **r = 0.297**
**HOMA-IR**	**ns**
**TTesto (ng/ml)**	**ns**
**Δ4A (ng/ml)**	**ns**
**DHEAS (μg/dl)**	**p = 0.031** **r = -0.143**
**SHBG (nmol/lt)**	**ns**
**Controls**	**Birth Weight (gr)**
**BMI (kg/m2)**	**ns**
**Weight Circumference (cm)**	**ns**
**HOMA-IR**	**ns**
**TTesto (ng/ml)**	**ns**
**Δ4A (ng/ml)**	**ns**
**DHEAS (μg/dl)**	**ns**
**SHBG (nmol/lt)**	**p = 0.021** **r = -0.234**

BMI: body mass index; HOMA-IR: homeostatic model assessment-insulin resistance; TTesto: Total Testosterone; Δ4A: androstenedione; DHEAS: dehydroepiandrosterone sulfate; SHBG: sex hormone binding globulin.

**Fig 1:**
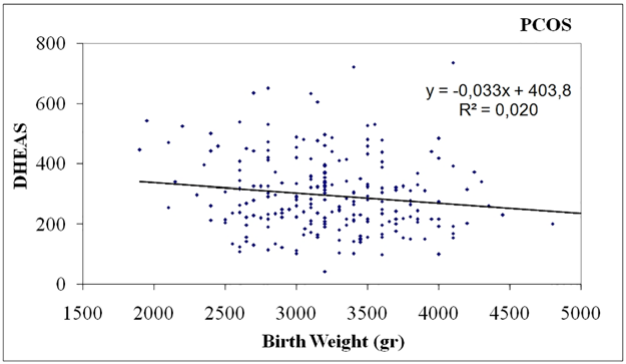
Negative correlation of birth weight with DHEAS levels in women with PCOS.

**Fig 2:**
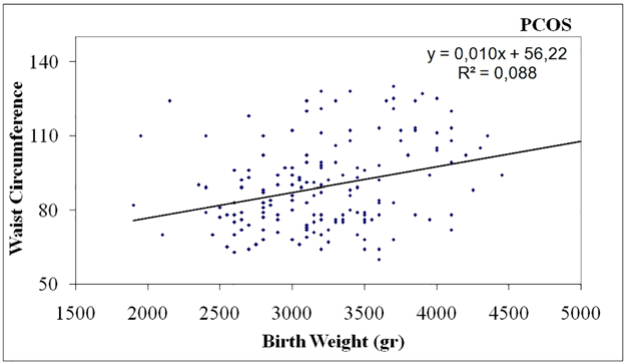
Positive correlation of birth weight with waist circumference in women with PCOS

**Fig 3:**
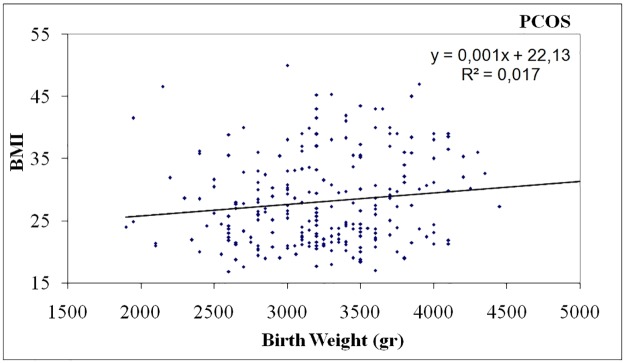
Positive correlation of birth weight with BMI in women with PCOS.

**Fig 4:**
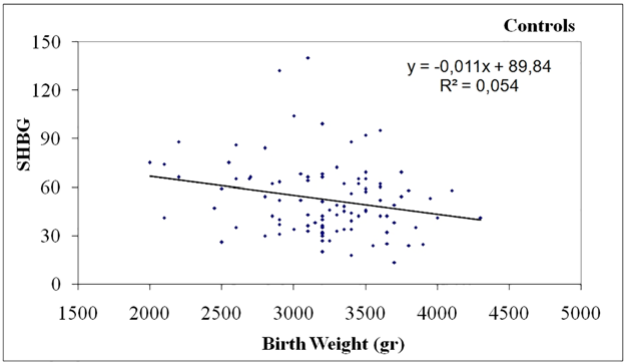
Negative correlation of birth weight with SHBG levels in Controls.

In PCOS women birth weight was negatively correlated with DHEA-S levels (p = 0.031, r = -0.143) (Table 2, Fig. 1), positively correlated with waist circumference (p <0.001, r = 0.297) (Table 2, Fig. 2) and body mass index (BMI) (p = 0.040, r = 0.132) (Table 2, Fig. 3). In controls, birth weight was negatively correlated with SHBG levels (p = 0.021, r = -0.234) (Table 2, Fig. 4).

The negative correlation of birth weight with DHEA-S levels in PCOS women was confirmed when we separated them in quartiles according to DHEA-S levels. Women from the highest quartile had statistically significant lower birth weight compared to the women from the lowest quartile (3118 ± 563 vs 3326 ± 497 gr, p = 0.033) (Table 3).

**Table 3:** Birth weight in women with PCOS according to DHEAS levels

	DHEAS Q1*	DHEAS Q2	DHEAS Q3	DHEAS Q4*
**Birth Weight (gr)**	**3326 ± 497**	**3298 ± 550**	**3230 ± 467**	**3118 ± 563**

Q: Quartile;

*Q1 vs Q4 p = 0.033, in all other comparisons p>0.05.

Women from both groups were further divided in 6 categories according to birth weight (A. <2.500 gr, B. 2.501–3.000 gr, C. 3.001–3.500 gr, D. 3.501–4.000 gr, E. 4.001–4.500 gr, F. > 4.500 gr) and percentage of women for each category was calculated. The distribution percentages in the above categories for both PCOS patients and controls are shown in Table 4. There was no statistically significant difference in the distribution percentages for each category between the two groups (A. 7% vs 7.9%, B. 26.8% vs 20.8%, C. 39.1% vs 48.5%, D. 21.4% vs 20.8%, E. 4.9% vs 2%, F. 0.8% vs 0%), (in all comparisons, p> 0.05) (Table 4).

**Table 4:** Percentages of patients and controls according to birth weight categories.

Birth Weight	Patients (*n = 243/288)	Controls (*n = 101/174)	p value
≤ **2.500 gr**	7%	7.9%	ns
**2.501–3.000 gr**	26.8%	20.8%	ns
**3.001–3.500 gr**	39.1%	48.5%	ns
**3.501–4.000 gr**	21.4%	20.8%	ns
**4.001–4.500 gr**	4.9%	2%	ns
**> 4.500 gr**	0.8%	0%	ns

## Discussion

In the present study we investigated the birth weight in women with PCOS and its possible correlation with clinical and biochemical characteristics of the syndrome. We did not find any differences neither in the mean birth weight nor in the distribution percentages of birth weight categories between PCOS women and normal controls.

Based on Barker’s intrauterine programming theory for chronic diseases [12] and the fact that insulin resistance is a cardinal feature of PCOS, a few studies have been conducted to investigate whether a causative link exists between birth weight and PCOS in adult life. An association between low or high birth weight and PCOS was found in some of them, but their results are constrained by the small number of participants [4, 13–14], investigation of specific group of patients, i.e. with precocious pubarche [13] and a deficient reproductive and metabolic phenotyping [5, 15–16].

Our findings are similar to the results of two large epidemiological studies. The first one, a well-designed and family-based study from the United States of America, showed that birth weight in families with PCOS defined by NIH criteria did not differ from the general population, even when it was corrected for gestational age. Furthermore, the investigators did not find any association of birth weight with reproductive and metabolic abnormalities both in women with PCOS and their relatives, males or females [8]. The second, a Finnish birth cohort study of 2007 women, showed no relationship between birth weight and self-reported PCOS symptoms of oligomenorrhea and hirsutism at the age of 31 years [7].

At variance, two recent studies with a large number of participants have shown that the presence of PCOS is not only associated with low but with increased birth weight as well. The first one was a retrospective birth cohort study of 948 singleton female babies from Australia. The results after adjusting for gestational age, suggested that two discrete fetal programming pathways, one related to high birth weight and the other to thinness at birth, are operating towards the manifestation of PCOS in adult life [5]. The second, a study from Denmark with the huge number of 523.757 participants had similar findings. Data were extracted from the Danish Civil Registration System and the Danish National Patient Register with the ICD codes and showed that the risk of PCOS was increased only in women born ≥4,500 g. Moreover, women born from mothers diagnosed with pre-gestational or gestational diabetes were at increased risk of PCOS, and this risk was inversely related to birth weight [6].

Similar results yielded from two other studies which investigated the link between birth weight and PCOS from another aspect i.e. by examining the birth weight of newborns of mothers with PCOS. The first one from Chile, found a significantly higher prevalence of babies small for gestational age in mothers with PCOS compared to newborns of normal women, matched for age and weight at the beginning of pregnancy [17]. The second one was a study from the Swedish Medical Birth Record comprising 1.195.123 singleton births between 1995 and 2007. A higher risk for being large for gestational age (1.39, 1.19 to 1.62) was documented among 3787 births from mothers with a PCOS diagnosis [18].

A possible explanation for these conflicting data could be that PCOS encompasses various phenotypic subtypes that are dictated by the parental genetic traits of the individual, the maternal contribution during fetal life and the adult environment. In large epidemiological studies, small subtypes (with low or high birth weight) probably yield no significant differences. Another explanation may be that the relation of birth weight and PCOS risk is not linear but a U-shaped, as it has been shown for insulin resistance and type 2 diabetes [19]. If this is the case then, a much larger number of patients is needed to prove this hypothesis.

An important finding of our study was that birth weight was negatively correlated with levels of DHEA-S and positively correlated with waist circumference and body mass index only in women with PCOS. To our knowledge, this association has not been found before, and it may be relevant to the fact that previous studies had not examined these parameters or included them in statistical analysis.

DHEA-S, produced by the adrenal glands, is an important pro-hormone of sex steroids from in utero and throughout life. Adrenal glands are vital organs for survival. Adverse endometrial conditions, which are reflected by low birth weight, may modulate the function of such organs, by changes in blood flow redistribution or in metabolic rates, a phenomenon known as developmental plasticity. This programming of “hyper-function” of the adrenal glands, which may be crucial for fetus survival in an adverse intrauterine environment, could lead to obesity and adrenal hyperandrogenism in childhood and possibly in adult life [20–21]. Several studies have shown that low birth weight is positively correlated with high DHEA-S levels in girls just before adrenarche until early adolescence [22–24]. Furthermore, a longitudinal study of a small group of lean girls with low birth weight and precocious pubarche from Spain showed that more than one third of them developed functional adrenal hyperandrogenism in adolescence [25].

Approximately 20–30% of women with PCOS have elevated levels of DHEA-S and this may be the only abnormality in circulating androgens in almost 10% of these women. Furthermore, an augmented adrenal response to exogenous ACTH stimulation [26–27] as well as an enhanced adrenal steroid production capacity up to menopause was documented in some women with PCOS [28–29]. However, taking into consideration that low birth weight (< 2500 gr) infants represent only 3–10% of total births in relevant studies [30] we can assume that other factors apart from birth weight such as heritability, insulin resistance or ovarian hyperandrogenemia could be responsible for high DHEA-S levels in women with PCOS [31]. Thus, those with low birth weight may constitute a subtype of the syndrome.

Obesity mainly central, is found in almost 50% of PCOS women and constitutes a characteristic feature of PCOS that can be encountered even in normal-weight patients. It can exacerbate insulin resistance as well as hyperandrogenemia and lead to an unfavorable metabolic profile [32–33]. Obesity and insulin resistance are strong heritable traits in PCOS families including male relatives [34–35]. We found that high birth weight in PCOS women was positively associated with BMI and waist circumference, a surrogate marker of central adiposity.

Epidemiological studies have documented that both low and high birth weight confer an increased risk for childhood and adult obesity [36–37]. Fetal under- or over-nutrition can have long term consequences for descendants’ health and continue the vicious cycle of obesity epidemic [38]. Thus, high birth weight in women with PCOS could have an additive effect on the development of obesity, insulin resistance and hyperandrogenism in adulthood [34].

In control women, the above correlations did not exist. However, birth weight in these women was negatively correlated with the levels of SHBG. SHBG has an important role in the regulation of bioavailable sex steroids, throughout human life (prenatal & antenatal) [39]. Genetic, hormonal and metabolic factors modulate SHBG production from the liver [21]. Moreover, SHBG was proved to have an inverse relation with obesity and insulin resistance and low SHBG levels are an independent risk factor for type 2 diabetes development [40]. In PCOS women SHBG levels are often low, independent of obesity and insulin resistance and may contribute to hyperandrogenic phenotype of the syndrome [34]. However, data upon birth weight correlation with SHBG in adult non PCOS women are very limited. In 592 premenopausal women from the Nurses’ Health Study no correlation was found between birth weight and SHBG levels [41]. In a study of young women from France, intrauterine growth restriction had no effect on SHBG levels [42]. A possible explanation for this finding is that SHBG levels may be the consequence of fetal programming for obesity and/or insulin resistance in adult women, not genetically determined to develop PCOS.

One limitation of our study could be the sample size. While it is large enough for a clinical study, it is relatively small from a general epidemiological point of view. Strengths of this study is the well-defined population of Caucasian women with PCOS and controls, that were all full-term born, with a similar socio-economic status.

In conclusion, in this cohort of women with PCOS birth weight was similar to age- and BMI-matched control women, probably due to a neutral net-effect of combined genetic, epigenetic and maternal factors upon birth weight. However, birth weight was associated with clinical and biochemical parameters of PCOS and may contribute to the phenotypic subtypes of the syndrome. Low birth weight was associated with adrenal hyperandrogenism, while high birth weight was associated with central obesity, both of which are features of the syndrome.
